# Use of Ultrasonography to Discriminate Psoriatic Arthritis from Fibromyalgia: A Post-Hoc Analysis of the ULISSE Study

**DOI:** 10.3390/jcm11010180

**Published:** 2021-12-29

**Authors:** Antonio Marchesoni, Pierluigi Macchioni, Stefania Gasparini, Carlo Perricone, Fabio Massimo Perrotta, Rosa Daniela Grembiale, Ettore Silvagni, Roberta Ramonda, Luisa Costa, Alen Zabotti, Giacomo Curradi, Giuliana Gualberti, Francesca Marando, Carlo Salvarani

**Affiliations:** 1 Rheumatology, Humanitas San Pio X, 20122 Milan, Italy; 2 Rheumatology Unit, Azienda USL-IRCCS di Reggio Emilia and Università di Modena and Reggio Emilia, 42123 Reggio Emilia, Italy; Pierluigi.Macchioni@ausl.re.it (P.M.); Carlo.Salvarani@ausl.re.it (C.S.); 3 Reumatologia, Dipartimento di Medicina Interna, Ospedale di Fabriano, ASUR Marche, 62012 Fabriano, Italy; gasparinistefania@libero.it; 4 Rheumatology, Department of Medicine and Surgery, University of Perugia, 06129 Perugia, Italy; carlo.perricone@gmail.com; 5 Dipartimento di Medicina e Scienze della Salute “Vincenzo Tiberio”, Università degli Studi del Molise, 86100 Campobasso, Italy; f.perrotta85@gmail.com; 6 Rheumatology Research Unit, Department of Health Sciences, “Magna Graecia” University, 88100 Catanzaro, Italy; rdgrembiale@unicz.it; 7 Rheumatology Unit, Department of Medical Sciences, University of Ferrara and Azienda Ospedaliero-Universitaria S. Anna, 44122 Ferrara, Italy; ettore.silvagni@edu.unife.it; 8 Rheumatology Unit, Department of Medicine-DIMED, University of Padova, 35131 Padova, Italy; roberta.ramonda@unipd.it; 9 Rheumatology Research Unit, Department of Clinical Medicine and Surgery, University of Naples Federico II, 80125 Naples, Italy; lv.costa@libero.it; 10 Department of Medical and Biological Science, Rheumatology Clinic, University of Udine, 33100 Udine, Italy; alen.zabotti@asufc.sanita.fvg.it; 11 AbbVie Srl, 00185 Rome, Italy; giacomo.curradi@abbvie.com (G.C.); giuliana.gualberti@abbvie.com (G.G.); Francesca.Marando@abbvie.com (F.M.)

**Keywords:** ultrasound, B mode, power Doppler, psoriatic arthritis, fibromyalgia syndrome, diagnosis

## Abstract

In psoriatic arthritis (PsA) patients with concomitant chronic widespread pain, the differential diagnosis with fibromyalgia syndrome (FMS) can be challenging. We evaluated whether ultrasound (US) examination of entheseal sites can distinguish pain from (PsA) enthesitis versus FMS. PsA and FMS patients underwent clinical evaluation and gray-scale (GS; B-mode) and power Doppler (PD) US examination of the entheses. At least one enthesis with GS- and PD-mode changes was found in 90% and 59.3% of PsA patients (*n* = 140) and 62.7% and 35.3% of FMS patients (*n* = 51), respectively. GS and PD identified changes in 49.5% and 19.2% of the 840 PsA entheses and 22.5% and 7.9% of the 306 FMS entheses, respectively. Receiver operating characteristic curve analysis showed an area under the curve of 0.77 and 0.66 for B- and PD-mode, respectively, 3.5 being the best cut-off GS-score to discriminate the two conditions. Multivariate regression showed that Achilles and proximal patellar tendon enthesitis (B-mode) were strongly associated with PsA (odds ratio, ~2). Principal component analysis (B-mode) confirmed that PsA patients have a higher number of involved entheses and patterns of entheseal involvement than FMS patients. US evaluation of the entheses may help differentiate chronic widespread pain from PsA versus FMS.

## 1. Introduction

The diagnosis of fibromyalgia syndrome (FMS) in the majority of patients is based on the presence of chronic widespread pain (CWP). In 2010, the American College of Rheumatology (ACR) proposed preliminary criteria for the diagnosis of FMS where, in addition to CWP, somatic symptoms were requested [1]. The 2016 revision confirmed that both CWP and somatic symptoms were needed to diagnose FMS and that “a diagnosis of fibromyalgia is valid irrespective of other diagnosis” [2]. These criteria do not include the tender point evaluation (a key feature of the 1990 ACR classification criteria) [3] that is still widely used in daily practice to diagnose this syndrome.

However, in cases of inflammatory musculoskeletal disorders with concomitant CWP, the diagnosis of FMS can be particularly challenging, as in the case of enthesitis typical of spondyloarthritis (SpA) [4]. Patients with SpA and CWP may present with polyenthesitis, FMS, or both. Furthermore, patients with unknown SpA characterized by polyenthesitis may easily be diagnosed incorrectly as having FMS. A recent study showed that compared with psoriatic arthritis (PsA), FMS was significantly associated with a high number of tender points and of somatic symptoms [5]. Although this finding may be helpful in daily practice, it is still very difficult to understand the precise role of FMS and polyenthesitis in the individual patient with PsA and CWP.

Musculoskeletal ultrasonography (US) has been shown to be a useful instrument for the assessment of SpA enthesitis [6,7] and to help distinguish between asymptomatic entheseal involvement in psoriasis, healthy controls, and PsA enthesitis [8]. US has also been found to provide a promising discriminating capacity between FMS and SpA in patients with inflammatory bowel disease [9]. In these studies, global US scores were significantly higher in PsA patients, and among the various US lesions, vascularization at bone-tendon instertion was the most specific for PsA. A recent study showed that hypoechogenicity, thickening, enthesophyte, erosion, and Doppler signal were the elementary lesions which best distinguished PsA patients from healthy controls [10]. An OMERACT study revealed that erosion, hypoechogenicity at the enthesis, Doppler at enthesis, and bursitis had the best intra-observer reliability, while bone irregularities, calcifications, and enthesophytes showed the lowest. As for the inter-observer reliability, the best performance was seen for enthesophytes and Doppler signal at insertion and the worst for thickened enthesis [11]. In a reliability analysis of elemental lesion erosions, bursae, and Doppler signal were found to have the highest values [12].

We recently evaluated the prevalence of clinical and US signs of enthesitis in patients with PsA compared with patients with psoriasis or FMS (the ULISSE study) [13]. There is a recognised difficulty in differentiating between psoriatic related polyenthesitis and fibromyalgia [4]. To address this, in the present post hoc analysis of the ULISSE study, we aimed to focus on the US features that may help distinguish the two conditions and on the patterns of aggregation of the US signals in the two study populations.

## 2. Materials and Methods

A thorough description of the methods employed in this study has been described previously [13]. The main methodological features of the present post hoc analyses are reported here.

### 2.1. Patients and Study Design

ULISSE was a cross-sectional, noninterventional, multicenter study performed in 10 Italian rheumatological centers and completed in 2013. Consecutive patients gave informed consent and were followed in accordance with the Italian Medicines Agency guidelines for noninterventional studies [14]; the ethical committees of all centers approved the study (First approval: prot n. 11/2011 dated 15 December 2011 of the EC “*Comitato Etico della provincia di Ferrara*”).

Inclusion criteria were that patients were ≥18 years and ≤65 years of age, were capable of giving informed consent, and had been diagnosed with either PsA (CASPAR criteria) [15] or FMS by a rheumatologist. Exclusion criteria were the presence of tendinitis due to overuse or physical stress (based on physician’s judgement), recent articular injury, any other inflammatory articular/connective tissue diseases, current treatment with systemic corticosteroids, current/past treatment with any disease-modifying antirheumatic drug (DMARD), local corticosteroid injections in the past 4 weeks, and any condition that, in the investigator’s opinion, would have jeopardized the patient’s ability to participate in the study.

### 2.2. Clinical and Ultrasound Assessment

Demographics, medical history, and disease characteristics were collected for each patient. Clinical general and articular examinations were performed by the investigator independent of the US evaluation. Enthesitis was measured using the Leeds Enthesitis Index (LEI) [16] and the Maastricht Ankylosing Spondylitis Enthesitis Score (MASES) [17].

The entheseal US assessment was performed by rheumatologists experienced in musculoskeletal US (sonographer). To standardize the US evaluation, all the sonographers attended a training meeting and received a booklet with standard US imaging. All the scan images were digitally recorded. All centers used the same US machine (ESAOTE My Lab70, Esaote SpA, Genoa, Italy) equipped with a broadband frequency transducer (6–18 MHz) and Doppler frequency (5.9–14.3 MHz). Each enthesis was scanned in grayscale (GS) and power Doppler (PD) mode.

The examined entheseal sites were the common extensor tendon insertion on the lateral epicondyle of the humerus, the quadriceps tendon, the proximal insertion of the patellar tendon, the proximal insertion of the medial collateral ligament of the knee, the Achilles tendon insertion on the calcaneus, and the plantar fascia insertion on the calcaneus, all of them bilaterally. In GS mode, the recorded changes were entheseal thickening, entheseal hypoechogenicity, peritendon hypoechogenicity, bony erosions, enthesophytes, and bursa enlargement. Each GS finding was scored 0 (absent) or 1 (present), for a maximum global score of 6 for each site. In addition, GS lesions were also classified as acute changes (hypoechogenicity and bursa enlargement) and chronic changes (thickening, bony erosions, and enthesophytes). In PD mode, blood flow was examined at the cortical bone insertion of the tendon, body of tendon, bursa, and junction between tendon and enthesis (pre-insertional area). PD changes were recorded as present or absent and scored according to the following number of vessels involved: 0 = none, 1 = 1–3 vessels, 2 = 4–5 vessels, and 3 = ≥5 vessels.

### 2.3. Statistical Analysis

The frequency and percentages of PD US findings are presented as involvement of at least one side for each paired entheseal site. The primary parameter was the rate of clinical and ultrasonographic signs of enthesitis. As the purpose of this analysis was to evaluate the results of the comparison between PsA and FMS, only the data of these two groups of patients are shown. The full description of the ULISSE results can be found elsewhere [13].

All variables have been summarized using descriptive analyses. Comparison between groups was performed using the Student *t* test (normal distribution) or Wilcoxon rank sum test (nonsymmetric, skewed distribution) for continuous variables, and chi-square test or Fisher exact test (as appropriate) for categorical variables. Whenever necessary, normality was assessed by means of the Shapiro–Wilk test. Backward stepwise logistic regression analysis was used to identify predictor variables for PsA diagnosis. The cut-off *p* values for inclusion in models were *p*_in_ = 0.05, and *p*_out_ = 0.10. Receiving-operator characteristic (ROC) curves were used to weigh the US scores and identify the most sensitive and specific cut-off values in favor of a PsA diagnosis. To explore distribution patterns of entheseal features in PsA versus FMS, we used principal component analysis (PCA) [18,19]. PCA was used to reduce the number of variables into a smaller number of new orthogonal variables, so-called principal components (PCs). Varimax rotation was used to extract the PCs because it maximizes the sum of the variances of the squared coefficients within each eigenvector, and the rotated axes remain orthogonal. Eigenvalues >1 were required to retain factors. Kaiser–Meyer–Olkin test of sampling adequacy and Bartlett’s test of sphericity were computed to establish the validity of the data set, at 1% level of significance. For all other analysis, *p* ≤ 0.05 was considered statistically significant. Statistical analyses were performed using SPSS statistical software, version 25.0 (SPSS, Chicago, IL, USA).

## 3. Results

### 3.1. Patient Characteristics

The numbers of patients with PsA and those with FMS enrolled in the ULISSE study were 140 and 51, respectively. In the PsA group, 70 patients (50%) were females; in the FMS group, 47 (92.2%) were females (*p* < 0.0001). Demographic and the main clinical features of the two groups are summarized in Table 1. As expected, BMI, ESR, and CRP values were significantly higher in patients with PsA.

### 3.2. Entheseal Clinical Findings

The median values (interquartile ranges, [IQRs]) of the MASES were 1 (0–6) and 7 (3–11) in PsA and FM patients, respectively (*p* < 0.001). The corresponding values for the LEI were 1 (0–3) and 3 (2–4) (*p* < 0.001). Of the two components of the LEI, swelling was never found in patients with FMS and was rarely seen in patients with PsA (median value 0 (0–0)); the LEI mean value related to swelling was higher in the PsA group (*p* = 0.019).

Table 2 shows the number of patients with the presence of objective signs of enthesitis in at least one of the paired sites for all the evaluated entheses. Tenderness was significantly more frequent in FMS than in PsA patients in most sites (FMS: 92.2%, PsA 66.4%, *p* = 0.0002). Plantar fascia insertions, Achilles tendons, quadriceps tendons, and both insertions of patellar tendons were similarly affected in the two groups. The percentages of entheses with signs of enthesitis were 23.2% and 45.6% in PsA and FMS patients, respectively (*p* < 0.0001).

### 3.3. Entheseal US Findings

At least one US abnormality was seen in 92.1% of PsA patients and also in a high percentage of FMS patients (74.5%, *p* = 0.0011). Changes by GS and PD mode were detected, respectively, in 90% and 59.3% of patients with PsA and 62.7% and 35.3% of patients with FMS (*p* < 0.0001 and *p* = 0.0033, respectively). In the PsA population, 451/840 pairs (53.7%) of examined entheses presented at least one US abnormality, versus 84/306 pairs (27.6%) in the FMS population (*p* < 0.00001). The GS and PD modes showed alterations in 49.5% and 19.2% of the entheses in the PsA group and 22.5% and 7.9% of the entheses in the FMS group, respectively (*p* < 0.0001 for both comparisons).

The median (IQR) PD global scores were 1 (0–5) and 0 (0–0) in patients with PsA and those with FMS, respectively (*p* < 0.0001). The corresponding values for the GS mode were 3 (1–6.75) and 1 (0–3) for the acute lesion (*p* < 0.0001) and 2 (0–5) and 0 (0–1) for the chronic lesion (*p* < 0.0001), respectively. The median (IQR) GS and PD scores at the examined entheses and the number and % patients with ≥1 lesion in each entheseal site using GS and PD are shown in Table 3. The PD score was significantly higher in patients with PsA only at the knee medial collateral ligament and proximal patellar tendon. In contrast, the GS score was significantly higher in patients with PsA in all sites, with the exception of the common extensor insertion on the epicondyle (Table 3).

The presence of PD signal is summarized in Table 4. In patients with FMS, it was undetectable or very rare in all sites. In these patients, the most common positivity was in the body of the tendons of the common extensor insertions on the epicondyles (about 10%). PD changes were more frequently observed in patients with PsA, but not enough to attain statistical significance. The most frequent presence of PD signal was in the pre-insertional area of the patella tendons (about 17%), followed by the body of the tendons of the common extensor insertions on the epicondyles (about 15%), and by cortical bone insertion of the medial collateral ligaments and of the Achilles tendons and the pre-insertional areas of the common extensor insertions on epicondyles and of the Achilles tendons (about 13%).

The frequency of the various GS changes is shown in Table 5. At the quadriceps tendon insertion, enthesophytes were significantly higher in the PsA population. At the patellar tendon and plantar fascia insertion, entheseal thickening and enthesophytes were found significantly more often in PsA patients. Alterations at the medial collateral ligament were uncommon in both study populations; nevertheless, entheseal hypoechogenicity was significantly more frequent in patients with PsA. In the Achilles tendon, entheseal thickening, entheseal hypoechogenicity, and enthesophytes were all significantly associated with PsA. With regard to the individual GS findings, enthesophyte was the feature that more often was able to discriminate between PsA and FMS patients, followed by entheseal thickening and entheseal hypoechogenicity.

### 3.4. Multivariate Logistic Regression Analysis

Stepwise logistic regression analysis was subsequently applied to identify potential predictors associated with a positive PsA diagnosis. Potential confounders, such as BMI and sex (significantly different between groups in the univariate analysis, Table S1), were included in models using a backward stepwise approach; variables that did not remain statistically significant were excluded. In the model with GS-mode scores, female sex was negatively associated with PsA, whereas BMI lost significance (Table S1). Global GS-mode score was positively associated with a PsA diagnosis (odds ratio [OR]: 1.24, 95% CI: 1.11–1.39, *p* < 0.0001); in subsequent models examining specific lesion involvement, the presence of a GS signal at the Achilles or patellar tendon and two GS lesions among entheseal hypoechogenicity, enthesophytes, and entheseal thickening were associated with a PsA diagnosis to a higher degree (OR: 2.07–2.26, 95% CI: 1.19–3.66) (Table S1).

In the model with PD score, female sex was again negatively associated with PsA, while PD global score was positively associated (OR: 2.77, 95% CI: 1.24–6.19, *p* = 0.013) (Table S2). BMI was also positively associated with PsA in models, including the PD score of specific districts instead of the global value. In this model, a positive PD signal at specific entheseal sites (medial collateral ligament or quadriceps tendon) yielded the greatest level of association for positive PsA diagnosis (OR: 4.06, 95% CI: 1.11–14.84, *p* = 0.034 and OR: 5.04, 95% CI: 1.06–23.9, *p* = 0.042, respectively) (Table S2).

### 3.5. ROC Analysis

**Figure 1:**
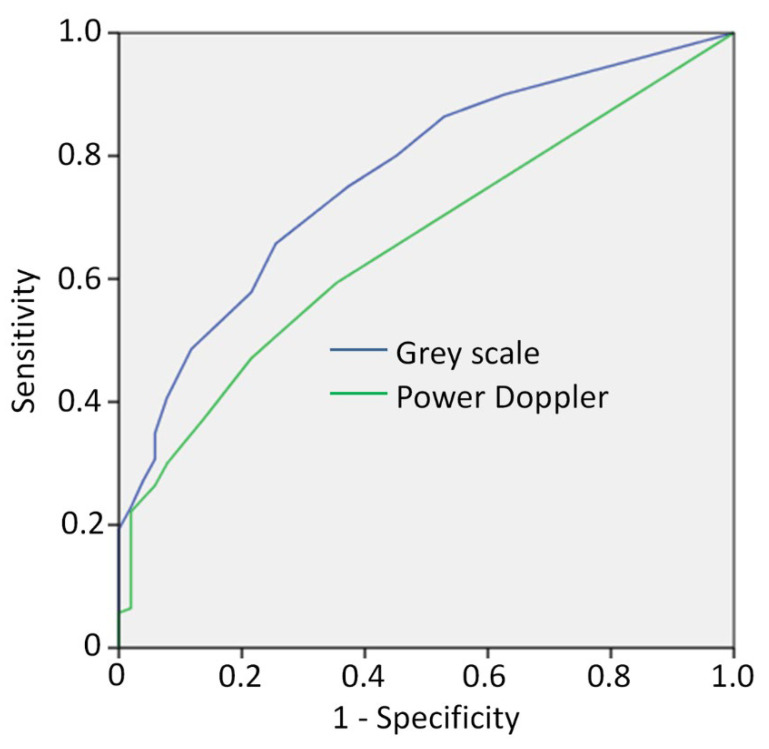
Receiver operating characteristic (ROC) curve to evaluate whether US scores could discriminate PsA from FMS using grey scale (dark blue line) and Power Doppler mode (green line).

We next used ROC curve analysis to evaluate whether US scores could discriminate PsA from FMS. ROC curves of GS and PD global scores showed an area under the curve (AUC) of 0.765 and 0.657, respectively, for the two modes (Figure 1). A global GS score of 3.5 was identified as the best cut-off to discriminate PsA from FMS (sensitivity = 0.75, specificity = 0.63, positive predictive value; PPV = 0.85). Specifically, the best performance for the GS was shown by the Achilles tendon (AUC = 0.714, 95% CI: 0.636–0.792) and proximal patellar tendon (AUC = 0.724, 95% CI: 0.650–0.798). The combination of the GS scores of these two sites yielded an AUC of 0.781 (95% CI: 0.713–0.849) with the best cut-off score of 2 (sensitivity = 0.86, specificity = 0.48 and PPV = 0.74). By PD mode, no entheseal site had an AUC > 0.6, which was deemed too low to permit further analysis. 

### 3.6. Principal Component Analysis (PCA)

PCA was next applied to explore distribution patterns of entheseal involvement and features in PsA and FMS. Only GS-mode lesions were included in this analysis. Using the global score in PsA, only one component (encompassing all entheseal sites) was found, explaining 43.2% of the total variance (Table S3). In FMS, three components (first: patellar tendon, plantar fascia, and quadriceps tendon; second: Achilles tendon; third: lateral epicondyle and medial collateral) accounted for 71.5% of the total variance (Table S3).

A PCA analysis performed using only three districts (Achilles, patellar, and quadriceps tendon) and excluding the lesions very uncommon in FMS (bone erosion and peritendon hypoechogenicity) yielded five components in PsA (65.7% of the variance) and three components in FMS (66.9% of the variance) (Table S4). Data dispersion was too wide to allow any interpretation. Using the same three sites but considering only hypoechogenicity, thickening, and enthesophyte, the PCA analysis yielded four clusters in PsA (70.2% of the variance) and three clusters in FMS (58.5% of the variance) (Table S5). The data distribution was wide even with this model, but with some interesting differences between the two diseases. In PsA, the first component was enthesophytes in the three sites, and the second was the other two lesions in the Achilles tendon (the two components explaining nearly 46% of the variance); in FMS, however, the same clusters were the second and the third component (nearly 36% of the variance). The same PCA analysis performed in the whole patient population showed a cluster distribution similar to that of PsA alone (Table S6). ROC analysis of the four components found by this evaluation showed that only the first cluster (enthesophyte in the three sites) was significantly associated with PsA, with a diagnostic accuracy of 65%. A further PCA analysis on the whole population, which also included bursa enlargement, showed five components accounting for 66% of the total variance (Table S7). The first cluster was composed of all lesions other than enthesophyte in the Achilles tendon, the second only of enthesophyte in this tendon, the third of thickening and hypoechogenicity at the patellar tendon and thickening at the quadriceps tendon. Interestingly, ROC analysis of these five components showed that the second and third clusters were significantly associated with PsA (Figure S1).

## 4. Discussion

The issues addressed by this subanalysis of the ULISSE study involved how the US examination of selected entheseal sites may help distinguish pain from psoriatic enthesitis versus FMS entheseal pain and how some specific US alterations aggregate in the two conditions. The two study populations showed the expected demographic and clinical differences, that is, more females and pain in the FMS group and higher BMI and serum inflammatory values in the PsA group. The low mean CRP values found in the PsA patients were presumably due to the exclusion of patients taking DMARDs and, as a result, the inclusion of cases with less active disease. Globally, US changes were significantly more frequent in patients with PsA than with FMS, both in the GS and the PD modes. However, in the FMS group, US abnormalities were seen in as many as 75% of patients and a PD signal in about 35% of patients. These figures are higher than those found in healthy populations. In a recent study, at least one US finding of active inflammation was seen in 30 out of 82 healthy subjects (34%) but a PD signal only in about 10% of the subjects [20]. A higher prevalence of US enthesitis in FMS patients than in healthy controls was also reported in a comparative study [21] where the mean values of the Madrid Sonographic Enthesitis Index were 7.39 ± 4.99 and 3.7 ± 3.22 in PsA and healthy subjects, respectively [22]. These data seem to suggest that some FMS patients might have an underlying inflammatory disease, but this will be an interesting issue to explore for future studies. The practical implication of our findings is that the mere presence of some US entheseal lesions seems to be of little value to differentiate PsA and FMS in the individual patient. In contrast, a distinction based on number, localization, and type of lesions proved to be more effective in identifying patients with PsA. The higher the number of GS abnormalities, the greater was the likelihood of having PsA, with a best cut-off point of global score of 3.5. Achilles and proximal patellar tendon GS involvement and having at least two GS lesions among enthesophyte, hypoechogenecity, and thickening in these two sites were indicative of PsA. This finding was confirmed by ROC analysis, where a combined GS score of 2 for these two sites was found to be the best cut-off discriminating the two diseases. PD global score was also independently associated with PsA, but to a lesser extent, and ROC analysis of this score could not differentiate the two diseases. The low prevalence of PD signal in the study population (19% and 8% of the entheses in PsA and FMS, respectively) likely accounted for the lack of a strong association of this inflammatory change with PsA. However, as this finding was rarely seen in FMS, its presence might be indicative of PsA, except for the elbow, in which virtually all of the US findings did not discriminate between the two diseases. The other US changes (peritendon hypoechogenicity, bony erosions, and bursa enlargements) were usually only seen in patients with PsA, but they were uncommon. Logistic analysis showed that BMI, which was significantly higher in patients with PsA, did not explain the difference in GS findings between the two study populations. However, we found that BMI was positively associated with PsA in models that included the PD score of specific districts. In a multicenter study of 225 ankylosing spondylitis, age, enthesophyte score, disease duration, and BMI were significantly associated with syndesmophytes in males by US examination [23].

The study of the patterns of distribution of the GS entheseal changes yielded some intriguing results. Lesions in all of the districts were detected as a cluster that was only found in PsA, although it included a minority of cases (43% of the variance). This result, however, confirms that an involvement of an elevated number of entheses is associated with PsA. In FMS, lesions were more dispersed, with an aggregation in three clusters. An analysis limited to the three sites more associated with PsA (Achilles, quadriceps, patellar tendon) and including the three more common lesions (hypoechogenicity, thickening, and enthesophyte) showed that enthesophytes in all of three sites or just in the Achilles tendon insertion discriminated PsA from FMS. Other aggregations were found, but not so strong as to provide useful clues for clinical practice. PCA analysis also found that there were patterns of entheseal involvement in FMS, although less consistent than in PsA. This result should be investigated in a larger FMS population to determine whether it could be of clinical relevance.

Other studies confirmed that US changes are consistently more frequent in patients with PsA, but as they are also present in those with FMS, the issue of defining distinguishing features that can be used in clinical practice remains critical [19,24]. No study on this topic has ever used a clustering approach.

## 5. Study Limitations

Our study has some limitations. The sample size was empirically determined, and it might have been underpowered for the statistical analysis relative to some US alterations. As patients with PsA taking DMARDs were not eligible, only patients with early or mild disease were enrolled. Nonetheless, the absence of DMARD treatment allowed to detect entheseal abnormalities without their interference. In contrast, nonsteroidal anti-inflammatory drugs were allowed and might have influenced the US findings in both study groups. The recently proposed criteria for FMS [3] were not available at the time of the study planning and could not be used to either classify patients with FMS or exclude the presence of this disorder in patients with PsA.

Another limitation is represented by patients with CWP as well as a genetic predisposition to psoriasis or SpA. Although these patients may have multiple enthesitis, they may be diagnosed as having FMS alone. Therefore, in cases of CWP, it may be worth examining factors predisposing to enthesitis [4].

The two study groups were not matched for sex and BMI. The higher BMI values in the patients with PsA may have influenced some of the US findings, although logistic regression analysis did not identify a significant impact of this variable. ULISSE was a multicenter study performed by nine sonographers, and intra-observer and inter-observer variation was not examined. Finally, patients aged ≥65 years were not included in this study to avoid introducing variables due to age, trauma, or overuse, and as such, our findings cannot be generalised to this elderly population.

## 6. Conclusions

This analysis suggests that entheseal US assessment may be helpful in differentiating PsA enthesitis from FMS tenderness. The number of entheses with US abnormalities, type of involved entheses, and type of US changes increase the probability of making a correct diagnosis. However, as US examination rarely provides definite answers, its integration with a thorough clinical evaluation is more than advisable and should always be taken into account. FMS might be concomitant to PsA enthesitis.

## Figures and Tables

**Table 1:** Demographics and main clinical features of patients with PsA and those with FMS.

Characteristic	PsA (*n* = 140)	FMS (*n* = 51)	*p* Value
Age, years	49 (40–58)	49 (42–57)	ns
Female, *n* (%)	70 (50)	47 (92.2)	**<0.0001**
White, *n* (%)	138 (98)	51 (100)	ns
Current smoker, *n* (%)	32 (23)	17 (33)	ns
Currently consumes alcohol, *n* (%)	63 (45)	19 (37)	ns
Disease duration, years ^a^	3 (1–8)	4 (2–10)	ns
BMI, kg/m^2^	25.5 (22.2–29.1)	23.4 (21.5–26.2)	**0.01**
DAS-28	3.07 (2.31–4.06)	2.56 (2.08–3.87)	ns
ESR, mm/h	12.5 (6.0–21.2)	7.0 (5.0–14.0)	**0.007**
CRP, mg/dL	0.30 (0.13–0.88)	0.20 (0.10–0.30	**0.001**

Data are presented as median (interquartile range) unless noted otherwise. BMI: body mass index, CRP: C-reactive protein, DAS-28: disease activity score in 28 joints, ESR: erythrocyte sedimentation rate; FMS: fibromyalgia syndrome; ns: not significant; PsA: psoriatic arthritis. ^a^ Since symptom onset. Statistically significant *p*-values values are indicated in bold.

**Table 2:** Frequency of signs of enthesitis in all of the examined entheseal sites in patients with PsA and those with FMS.

Entheseal Site	PsA (*n* = 140)		FMS (*n* = 51)		*p* ^b^	*p* Value ^b^
	Swelling ^a^	Tenderness	Swelling ^a^	Tenderness		
Epicondyle	3 (2.14)	54 (38.57)	0	39 (76.47)	ns	**0.0365**
Medial condyle of the femur	2 (1.43)	29 (20.71)	0	26 (50.98)	ns	**0.0001**
Achilles tendon	12 (8.57)	41 (29.29)	0	13 (25.49)	ns	ns
1st costochondral joint	na	32 (22.86)	na	33 (64.71)	na	**0.0000**
7th costochondral joint	na	29 (20.71)	na	30 (58.82)	na	**0.0000**
Posterior superior spine	na	35 (25)	na	25 (49.02)	na	**0.0028**
Anterior superior spine	na	18 (12.86)	na	20 (39.22)	na	**0.0001**
Iliac crest	na	23 (16.43)	na	21 (41.18)	na	**0.0000**
5th lumbar spinous process	na	36 (27.14)	na	26 (54.90)	na	**0.0000**
Quadriceps tendon	na	26 (18.57)	na	11 (21.57)	na	ns
Proximal insertion patellar tendon	na	24 (17.14)	na	14 (27.45)	na	ns
Distal insertion patellar tendon	na	19 (13.57)	na	13 (25.49)	na	ns
Insertion of the plantar fascia	na	31 (22.14)	na	13 (25.49)	na	ns

Data are presented as number (%) of patients with ≥1 involved site. FMS: fibromyalgia syndrome, na: not applicable, ns: not significant, PsA: psoriatic arthritis. ^a^ Swelling was evaluated only in the sites included in the Leeds Enthesitis Index. ^b^ *p* values for swelling only. Statistically significant p-values values are indicated in bold.

**Table 3:** GS- and PD-US global scores at the examined entheseal sites in the two study populations.

Entheseal Site	GS-US Score			PD-US Score		
	PsA (*n* = 140)	FMS (*n* = 51)	*p* Value	PsA (*n* = 140)	FMS (*n* = 51)	*p* Value
Common extensor insertion on the epicondyle	0 (0–2) 50 (35.7)	0 (0–0) 12 (23.5)	0.077	0 (0–0.75) 35 (25)	0 (0–0) 8 (15.7)	0.136
Quadriceps tendon	2 (0–3) 97 (69.3)	0 (0–2) 21 (41.2)	**0.001**	0 (0–0) 18 (12.9)	0 (0–0) 2 (3.9)	0.077
Patellar tendon	1 (0–2) 89 (63.6)	0(0–0) 12 (23.5)	**<0.0001**	0 (0–0) 25 (17.9)	0 (0–0) 2 (3.9)	**0.016**
Medial collateral ligament	0 (0–0) 28 (20)	0 (0–0) 1 (1.96)	**0.002**	0 (0–0) 30 (21.4)	0 (0–0) 3 (5.9)	**0.010**
Achilles tendon	2 (0–3) 98 (70)	0 (0–1) 17 (33.33)	**<0.0001**	0 (0–0) 33 (23.6)	0 (0–0) 6 (11.8)	0.065
Plantar fascia insertion on the calcaneus	0 (0–0) 53 (37.9)	0 (0–0) 6 (11.76)	**0.001**	0 (0–0) 2 (1.4)	0 (0–0) 0 (0)	0.392

Data are presented as median (interquartile range) and number (%) of patients with ≥1 lesion in each entheseal site. FMS: fibromyalgia syndrome, GS: gray scale, PD: power Doppler, PsA: psoriatic arthritis, US: ultrasound. Statistically significant p-values values are indicated in bold.

**Table 4:** Frequency of the presence of PD-US changes at the examined entheseal sites in patients with PsA (*n* = 140) and those with FMS (*n* = 51).

Entheseal Site	Cortical Bone Insertion			Pre-Insertional Area			Body of Tendon			Bursa		
	PsA	FMS	*p* Value	PsA	FMS	*p* Value	PsA	FMS	*p* Value	PsA	FMS	*p* Value
Common extensor insertion on the epicondyle	15 (10.7)	3 (5.9)	ns	18 (12.9)	3 (5.9)	ns	21 (15)	5 (9.8)	ns	0	1 (2)	ns
Quadriceps tendon	10 (7.1)	1 (2)	ns	8 (5.7)	2 (3.9)	ns	7 (5)	0	ns	5 (3.6)	1 (2)	ns
Patellar tendon	13 (9.3)	1 (2)	ns	24 (17.1)	3 (5.9)	ns	7 (5)	1 (2)	ns	2 (1.4)	0	ns
Medial collateral ligament	18 (12.9)	1 (2)	ns	8 (5.7)	1 (2)	ns	12 (8.6)	1 (2)	ns	1 (0.7)	0	ns
Achilles tendon	18 (12.9)	3 (5.9)	ns	18 (12.9)	1 (2)	ns	14 (10)	2 (3.9)	ns	11 (7.9)	1 (2)	ns
Plantar fascia insertion on the calcaneus	0	0	ns	1 (0.7)	0	ns	1 (0.7)	0	ns	0	0	ns

Data are presented as number (%) of patients with ≥1 involved site. FMS: fibromyalgia syndrome, ns: not significant, PD: power Doppler, PsA: psoriatic arthritis, US: ultrasound.

**Table 5:** Frequency of the various GS-US changes at the examined entheseal sites in patients with PsA (*n* = 140) and those with FMS (*n* = 51).

**Entheseal Site**	**Entheseal** **Thickening**			**Entheseal** **Hypoechogenicity**			**Peritenon** **Hypoechogenicity**		
	**PsA**	**FMS**	***p* Value**	**PsA**	**FMS**	***p* Value**	**PsA**	**FMS**	***p* Value**
Common extensor insertion on the epicondyle	21 (15)	3 (5.9)	ns	26 (18.6)	8 (15.7)	ns	7 (5)	1 (2)	ns
Quadriceps tendon	41 (29.3)	9 (17.3)	ns	34 (30.7)	15 (29.4)	ns	8 (5.7)	0	ns
Patellar tendon	49 (35)	4 (7.8)	**0.003**	49 (35)	4 (7.8)	ns	11 (7.9)	2 (3.9)	ns
Medial collateral ligament	9 (6.4)	1 (2)	ns	19 (13.6)	0	**0.006**	5 (3.6)	0	ns
Achilles tendon	32 (22.9)	2 (3.9)	**0.003**	40 (28.6)	4 (7.8)	**0.003**	12 (8.6)	1 (2)	ns
Plantar fascia insertion on the calcaneus	30 (21.4)	4 (7.8)	**0.014**	25 (17.9)	2 (3.9)	ns	5 (3.6)	0	ns
	**Bony Erosion**			**Enthesophyte**			**Enlarged Bursa**		
**Entheseal Site**	**PsA**	**FMS**	***p* Value**	**PsA**	**FMS**	***p* Value**	**PsA**	**FMS**	***p* Value**
Common extensor insertion on the epicondyle	12 (8.9)	4 (7.8)	ns	31 (22.1)	6 (11.8)	ns	1 (0.7)	1 (2)	ns
Quadriceps tendon	4 (2.9)	0	ns	63 (45)	8 (15.7)	**<0.0001**	11 (7.9)	3 (5.9)	ns
Patellar tendon	9 (6.4)	1 (2)	ns	29 (20.7)	4 (7.8)	**<0.0001**	11 (7.9)	1 (2)	ns
Medial collateral ligament	9 (6.4)	0	ns	6 (4.3)	0	ns	2 (1.4)	0	ns
Achilles tendon	15 (10.7)	1 (2)	ns	66 (47.1)	13 (25.5)	**0.007**	25 (17.9)	5 (9.8)	ns
Plantar fascia insertion on the calcaneus	5 (3.6)	0	ns	19 (13.6)	2 (3.9)	**0.03**	0	0	ns

Data are presented as number (%) of patients with ≥1 involved site. FMS: fibromyalgia syndrome, GS: gray scale, ns: not significant, PsA: psoriatic arthritis, US: ultrasound. Statistically significant p-values values are indicated in bold. ns = non-statistically significant difference.

## Data Availability

Data can be made available from the corresponding author upon request.

## Supplementary Materials

The following are available online at https://www.mdpi.com/article/10.3390/jcm11010180/s1. Table S1: Stepwise logistic regression analysis to examine predictors of PsA diagnosis using B-mode. Table S2: Stepwise logistic regression analysis to examine predictors of PsA diagnosis using PD-mode. Table S3: Principal component analysis results in FMS and PsA: PCA1. Table S4: Principal component analysis results in FMS and PsA: PCA2. Table S5: Principal component analysis results in FMS and PsA: PCA3. Table S6: Principal component analysis results in all patients: PCA4. Table S7: Principal component analysis results in all patients: PCA5. Figure S1: PCA on whole population eb, eh, et, ent at AT, PT, QT districts.
