# Supplementary material for: Use of Ultrasonography to Discriminate Psoriatic Arthritis from Fibromyalgia: A Post-Hoc Analysis of the ULISSE Study
Source: J Clin Med. 2021 Dec 29;11(1):180. doi: 10.3390/jcm11010180 (PMC8745640; doi:10.3390/jcm11010180)
Supplement: Supplementary file 1 [file jcm-11-00180-s001.zip › jcm-1473755-supplementary.pdf]

Supplementary Material:

Table S1. Stepwise logistic regression analysis to examine predictors of PsA diagnosis using B-mode.

Model	Variables	*B*-coefficient	OR (95% CI)	p-value
1	BMI	0.039	1.04 (0.96-1.13)	0.347
	Sex (female)	-2.25	1.11 (0.035-0.32)	**<0.0001**
	Score B_tot	0.22	1.24 (1.11-1.39)	**<0.0001**
	Constant	0.7	2.01	0.547
2	Sex (female)	-2.27	0.10 (0.034-0.31)	**<0.0001**
	Score B_tot	0.23	1.25 (1.13-1.4)	**<0.0001**
	Constant	1.65	5.19	**0.004**
3	Sex (female)	-2.22	0.11 (0.035-0.34)	**<0.001**
	BMI	0.028	1.03 (0.95-1.12)	0.52
	B-mode_AT	0.45	1.57 (1.12-2.19)	**0.009**
	B-mode_PT	0.61	1.85 (1.2-2.84)	**0.006**
	Constant	0.96	2.62	0.42
4	Sex (female)	-2.23	0.11 (0.035-0.33)	**<0.001**
	B-mode_AT	0.45	1.57 (1.13-2.19)	**0.008**
	B-mode_PT	0.65	1.91 (1.25-2.92)	**0.003**
	Constant	1.64	5.17	**0.003**
5	Sex (female)	-2.35	0.095 (0.031-0.29)	**<0.001**
	BMI	0.045	1.05 (0.96-1.14)	0.28
	B-mode_AT_eh_ent	0.81	2.24 (1.43-3.49)	**<0.001**
	B-mode_PT_eh_et	0.73	2.07 (1.19-3.59)	**0.010**
	Constant	0.66	1.93	0.58
6	Sex (female)	-2.37	0.094 (0.031-0.29)	**<0.001**
	B-mode_AT_eh_ent	0.82	2.26 (1.45-3.51)	**<0.001**
	B-mode_PT_eh_et	0.76	2.13 (1.24-3.66)	**0.006**
	Constant	1.78	5.95	**0.001**

AT = Achilles tendon insertion on the calcaneus, BMI = body mass index, CI = confidence interval, eh = entheseal hypoechogenicity, ent = entesophytes, et = entheseal thickening, OR = odds ratio, PT = proximal insertion of the patellar tendon

Table S2. Stepwise logistic regression analysis to examine predictors of PsA diagnosis using PD-mode.

Model*	Variables	*B*-coefficient	OR (95% CI)	p-value
1	Sex (female)	-2.39	0.09 (0.03-0.27)	**<0.0001**
	BMI	0.066	1.07 (0.99-1.15)	0.085
	Score PD_01(1)	1.02	2.77 (1.24-6.19)	**0.013**
	Constant	0.79	2.22	0.466
2	Sex (female)	-2.31	0.09 (0.032-0.28)	**<0.0001**
	BMI	0.08	1.09 (1.00-1.17)	**0.029**
	PD_MC_DIC (1)	1.4	4.06 (1.11-14.84)	**0.034**
	Constant	0.54	1.71	0.62
3	Sex (female)	-2.48	0.08 (0.028-0.25)	**<0.0001**
	BMI	0.09	1.09 (1.01-1.17)	**0.023**
	PD_QT_DIC (1)	1.62	5.04 (1.06-23.9)	**0.042**
	Constant	0.58	1.78	0.6

AT = Achilles tendon, BMI = body mass index, CI = confidence interval, DIC = dichotomous data, PD = power Doppler, MC = proximal insertion of the medial collateral ligament of the knee OR = odds ratio, PT = patella, QT = quadriceps tendon

*(0 = score of 0-1, 1 for score ≥2).

Table S3. Principal component analysis results in FMS and PsA: PCA1.

	PSA	FMS*		
Explained variance (%)	43.19		71.5	
Variance explained for each component (%)	43.19	29.99	22.87	18.67
Component	1	1	2	3
B-mode_AT	0.750		0.915	
B-mode_LAT_EPI	0.630			0.700
B-mode_MC	0.659			0.855
B-mode_PT	0.592	0.646		
B-mode_PF	0.668	0.686		
B-mode_QT	0.632	0.871		

Method of rotation: Varimax Kaiser normalization.
*Convergence per rotation followed in 4 iterations.

AT=Achilles tendon insertion on the calcaneus, LAT_EPI = lateral epicondyle, MC = medial collateral, PT=proximal insertion of the patellar tendon, PF = plantar fascia and QT=quadriceps tendon;

Table S4. Principal component analysis results in FMS and PsA: PCA2

	PsA*					FMS**			
Explained variance (%)	65.67					66.9			
Variance explained for each component (%)	21.79	15.33	10.31	9.47	8.78	21.33	18.98	14.63	11.95
Component	1	2	3	4	5	1	2	3	4
B-mode_AT_eb	0.646								0.812
B-mode_AT_eh	0.786						0.700		
B-mode_AT_et	0.831						0.831		
B-mode_AT_ent		0.803						0.644	
B-mode_PT_eb					0.549		0.872		
B-mode_PT_eh			0.794			0.882			
B-mode_PT_et			0.746						0.808
B-mode_PT_ent		0.647						0.775	
B-mode_QT_eb				0.871		0.811			
B-mode_QT_eh				0.660		0.683			
B-mode_QT_et					0.730	0.684			

B-mode_QT_ent		0.796						0.722	

Method of rotation: Varimax Kaiser normalization.
*Convergence per rotation followed in 5 iterations.
**Convergence per rotation followed in 6 iterations.

AT=Achilles tendon insertion on the calcaneus, EB=enlarged bursa, EH=entheseal hypoechogenicity, ENT=enthesophytes, ET=entheseal thickening, PT=proximal insertion of the patellar tendon, and QT=quadriceps tendon

Table S5. Principal component analysis results in FMS and PsA: PCA3

			PsA*			FMS**	
Explained variance (%)			70.2			58.5	
Variance explained for each component (%)	26.59	19.27	12.63	11.7	22.62	19.42	16.42
Component	1	2	3	4	1	2	3
B-mode_AT_eh		0.864					0.780
B-mode_AT_et		0.896					0.761
B-mode_AT_ent	0.784					0.591	
B-mode_PT_eh				0.905	0.845		
B-mode_PT_et			0.483	0.491			
B-mode_PT_ent	0.669					0.766	
B-mode_QT_eh			0.611		0.647		
B-mode_QT_et			0.883		0.818		
B-mode_QT_ent	0.791					0.773	

Method of rotation: Varimax Kaiser normalization.
*Convergence per rotation followed in 5 iterations.
**Convergence per rotation followed in 6 iterations.

AT=Achilles tendon insertion on the calcaneus, EB=enlarged bursa, EH=entheseal hypoechogenicity, ET=entheseal thickening, ENT=enthesophytes, PT=proximal insertion of the patellar tendon, and QT=quadriceps tendon

Table S6. Principal component analysis results in all patients: PCA4

			PsA	
Explained variance (%)			70.14	
Variance explained for each component (%)	27.15	19.69	11.78	11.52

Component	1	2	3	4
B-mode_AT_eh		0.853		
B-mode_AT_et		0.906		
B-mode_AT_ent	0.794			
B-mode_PT_eh				0.898
B-mode_PT_et				0.487
B-mode_PT_ent	0.677			
B-mode_QT_eh			0.659	
B-mode_QT_et			0.876	
B-mode_QT_ent	0.803			

Method of rotation: Varimax Kaiser normalization.
Convergence per rotation followed in 5 iterations.

AT=Achilles tendon insertion on the calcaneus, EB=enlarged bursa, EH=entheseal hypoechogenicity, ET=entheseal thickening, ENT=enthesophytes, PT=proximal insertion of the patellar tendon, and QT=quadriceps tendon

Table S7. Principal component analysis results in all patients: PCA5

	All patients				
Explained variance (%)			66.03		
Variance explained for each component (%)	22.68	14.98	10.53	9.24	8.6
Component	1	2	3	4	5
B-mode_AT_eb	0.668				
B-mode_AT_eh	0.769				
B-mode_AT_et	0.786				
B-mode_AT_ent		0.805			
B-mode_PT_eb					0.609
B-mode_PT_eh			0.634		
B-mode_PT_et			0.816		
B-mode_PT_ent		0.660			
B-mode_QT_eb				0.850	
B-mode_QT_eh				0.760	
B-mode_QT_et			0.542		
B-mode_QT_ent		0.804			

Method of rotation: Varimax Kaiser normalization.
Convergence per rotation followed in 7 iterations.

AT=Achilles tendon insertion on the calcaneus, EB=enlarged bursa, EH=entheseal hypoechogenicity, ET=entheseal thickening, ENT=enthesophytes, PT=proximal insertion of the patellar tendon, and QT=quadriceps tendon

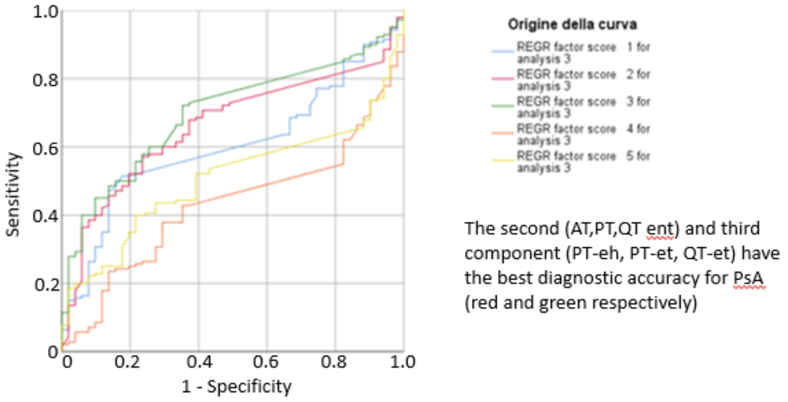

Variables/results from test	Area	Standard Error[a]	P-value	95% confidence intervals	
				Lower limit	Upper limit
REGR factor score 1 for analysis 3	0.604	0.042	0.028	0.522	0.686
REGR factor score 2 for analysis 3	0.662	0.040	0.001	0.582	0.741
REGR factor score 3 for analysis 3	0.699	0.039	0.000	0.624	0.775
REGR factor score 4 for analysis 3	0.431	0.042	0.145	0.348	0.514
REGR factor score 5 for analysis 3	0.508	0.042	0.866	0.427	0.589

Results from REGR factor score 1 for analysis 3, REGR factor score 2 for analysis 3, REGR factor score 3 for analysis 3, REGR factor score 4 for analysis 3, REGR factor score 5 for analysis 3 have at least a correlation between the group with positive effect and group with negative effects. Statistics can be distorted.

a. Based on non-parametric hypothesis

b. Null hypothesis: true, area = 0.5

Figure S1. PCA on whole population eb, eh, et, ent at AT, PT, QT districts.
